# Complete mitochondrial genome of the freshwater fish, *Carassioides acuminatus* (Cypriniformes, cyprinidae)

**DOI:** 10.1080/23802359.2017.1310602

**Published:** 2017-04-09

**Authors:** Shuli Zhu, Xinhui Li, Jiping Yang, Yuefei Li, Fangcan Chen, Jie Li, Zhi Wu

**Affiliations:** a Pearl River Fisheries Research Institute, Chinese Academy of Fishery Sciences, Guangzhou, China;; b Experimental Station for Scientific Observation on Fishery Resources and Environment in the Middle and Lower Reaches of Pearl River, Ministry of Agriculture The People’s Republic of China, Guangzhou, China

**Keywords:** *Carassioides acuminatus*, mitochondrial genome, Cyprinidae

## Abstract

In this study, the complete mitochondrial genome of *Carassioides acuminatus* was first sequenced and annotated. The entire mitogenome is 16,579bp in length, which consists of 13 protein-coding genes (PCGs), 22 transfer RNA genes, 2 ribosomal RNA genes, and a control region (D-loop). The overall nucleotide composition of the *C. acuminatus* mitochondrial genome shows an obvious anti-G bias. The accuracy of the fresh sequences was verified by phylogenetic analysis. The complete mitochondrial genome of *C. acuminatus* is useful to population genetics and molecular systematics.

*Carassioides acuminatus* (*C. acuminatus*) is a small-to moderate-sized freshwater economic fish belonging to the family Cyprinidae, order Cypriniformes (Zheng 1989). It is mainly distributed in the Pearl River and Hainan Island water system in China. As the *C. acuminatus* geographical distribution range is narrow, few studies have been reported (Chunxing et al. 2014). Due to its haploid nature, limited recombination, maternal inheritance, and rapid evolutionary rate, the mitochondrial DNA has now been widely used for studying population genetics, phylogeography and phylogeny and species identification. Consequently, it was necessary to determine the complete mitochondrial genome of the *C. acuminatus* and identify its phylogenetic relationships with the closely related species for the sustainable utilization of the *C. acuminatus* fishery resource.

In this study, the sample of the *C. acuminatus* was obtained from Lingao section (19°34′–20°02′ N, 109°3′–109°53′ E) in the Wenlan River, Hainan, China. It was stored in the Pearl River Fisheries Research Institute, Chinese Academy of Fishery Sciences, Guangzhou, China with the sample number CAWL2014. The complete mitochondrial genome of the *C. acuminatus* was sequenced using Illumina-based *de novo* transcriptome technology and annotated using bioinformatic tools (Laslett & Canbäck 2008; Tamura et al. 2013). The mitochondrial genome of *C. acuminatus* was 16,579 bp in length and had been deposited in GenBank with accession number of KX602324. In comparison with the other fish (Zhang et al. 2016), the mitogenome of *C. acuminatus* share the same organization consisting of 13 protein-coding genes(PCGs), 22 transfer RNA (tRNA) genes, 2 ribosomal RNA (rRNA) genes, and a putative control region. Except for *ND6* and eight tRNA genes (*tRNA-Gln*, *tRNA-Ala*, *tRNA-Asn*, *tRNA-Cys*, *tRNA-Tyr*, *tRNA-Ser*, *tRNA-Pro*, and *tRNA-Glu*), which are encoded on the light strand (L-strand), the remaining genes are encoded on the heavy strand (H-strand). The overall nucleotide composition of *C. acuminatus* mitochondrial genome is A:34.05%, T:32.76%, G:13.36%, and C:19.83%, with the A + T content of 66.81%, showing an obvious anti-G bias in accordance with the mitochondrial genomes of other teleost species (Norfatimah et al. 2014; Xie et al. 2016). Among all 13 protein-coding genes, we found that most protein-coding genes for *C. acuminatus* share the common initiation codon ATG, while only *COXI* gene which start from GTG instead of ATG. Besides, incomplete termination codons (T or TA) were also found in six genes (*ND2*, *COXII*, *COXIII*, *ND3*, *ND4*, and *Cytb*), which may be completed by polyadenylation of the RNA messenger after cleavage (Nardi et al. 2001) (Table 1).

**Table 1.** Characteristics of the mitochondrial genome of *C. acuminatus*.

		Position				Codon			
Locus	Strand	From	To	Size Nucleotide (bp)	Amino acid	Start	Stop	Anti-codon	Intergenic nucleotide
*tRNA-Phe*	H	1	69	69				GAA	0
*12S-rRNA*	H	70	1023	954					0
*tRNA-Val*	H	1024	1095	72				TAC	0
*16S-rRNA*	H	1096	2773	1678					0
*tRNA-Leu*	H	2774	2849	76				TAA	1
*ND1*	H	2851	3825	975	324	ATG	TAA		4
*tRNA-Ile*	H	3830	3901	72				GAT	−2
*tRNA-Gln*	L	3900	3970	71				TTG	1
*tRNA-Met*	H	3972	4040	69				CAT	0
*ND2*	H	4041	5085	1045	348	ATG	T		0
*tRNA-Trp*	H	5086	5156	71				TCA	1
*tRNA-Ala*	L	5158	5226	69				TGC	1
*tRNA-Asn*	L	5228	5300	73				GTT	35
*tRNA-Cys*	L	5336	5402	67				GCA	−1
*tRNA-Tyr*	L	5402	5472	71				GTA	1
*COXI*	H	5474	7024	1551	516	GTG	TAA		0
*tRNA-Ser*	L	7025	7095	71				TGA	3
*tRNA-Asp*	H	7099	7170	72				GTC	12
*COXII*	H	7183	7873	691	230	ATG	T		0
*tRNA-Lys*	H	7874	7949	76				TTT	1
*ATP8*	H	7951	8115	165	54	ATG	TAG		−7
*ATP6*	H	8109	8792	684	227	ATG	TAA		−1
*COXIII*	H	8792	9576	785	261	ATG	TA		0
*tRNA-Gly*	H	9577	9648	72				TCC	0
*ND3*	H	9649	9997	349	116	ATG	T		0
*tRNA-Arg*	H	9998	10067	70				TCG	0
*ND4L*	H	10068	10364	297	98	ATG	TAA		−7
*ND4*	H	10358	11738	1381	460	ATG	T		0
*tRNA-His*	H	11739	11807	69				GTG	0
*tRNA-Ser*	H	11808	11876	69				GCT	1
*tRNA-Leu*	H	11878	11950	73				TAG	3
*ND5*	H	11954	13777	1824	607	ATG	TAA		−4
*ND6*	L	13774	14295	522	173	ATG	TAG		0
*tRNA-Glu*	L	14296	14364	69				TTC	5
*Cytb*	H	14370	15510	1141	380	ATG	T		0
*tRNA-Thr*	H	15511	15582	72				TGT	−1
*tRNA-Pro*	L	15582	15651	70				TGG	0
*D-loop*	H	15652	16579	928					0

There are seven regions of gene overlap ranging from 1 to 7 bp and 13 intergenic spacer regions ranging from 1 to 35 bp with the longest intergenic region appeared between tRNA-Asn and tRNA-Cys. Overlapped gene was believed to be associated with the transition from RNA to DNA synthesis (Hixson et al. 1986) (Table 1).

Similarly to other mitochondrial genomes, the two ribosomal RNA genes (*12S rRNA* and *16S rRNA*) are located between *tRNA-Phe* and *tRNA-Leu* within *C. acuminatus* mitogenome, and separated by *tRNA-Val*. Besides, our analysis indicated that 22 tRNA genes varying from 67 to 76 bp are interspersed throughout the genome. The control region (D-loop) is 928 bp in length which is located between the *tRNA-Pro* and *tRNA-Phe* genes, as generally shown in most vertebrate mitochondrial genome (Liu & Yang 2013; Quan et al. 2013) (Table 1).

The phylogenetic tree was constructed on the basis of the complete mitogenome sequences from *C. acuminatus* and other 15 closely related species in the GenBank database. A neighbour-joining tree was constructed by using MEGA 5.1 Program. According to the established phylogenetic tree, we confirm that the *C. acuminatus* is much closer to Carassius, which coincides to the morphological taxonomy (Figure 1).

**Figure 1.**
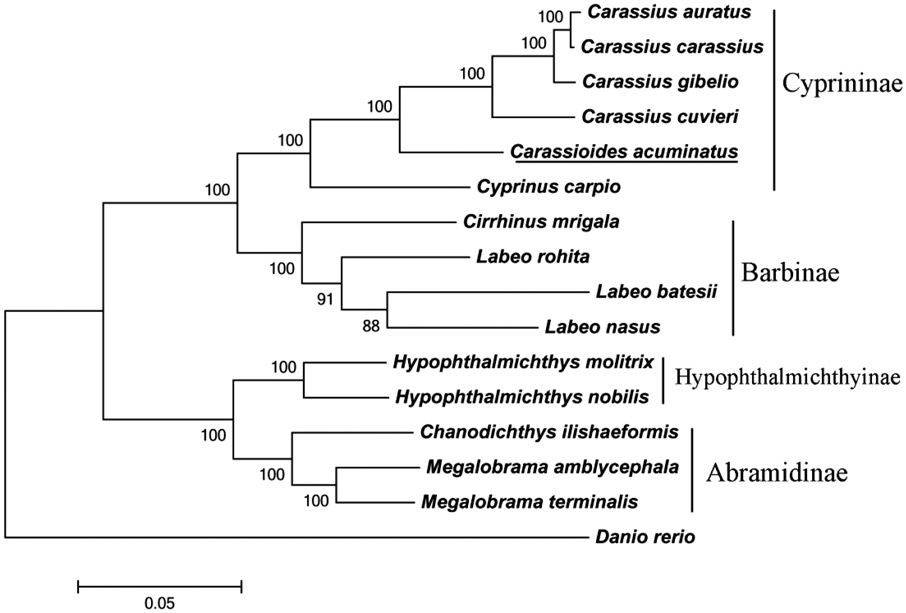
The phylogenetic tree based on 16 related mitochondrial genomes. The accession numbers of the species are *C. acuminatus* (KX602324.1), *Carassius auratus* (AB111951.1), *C. carassius* (AY714387.1), *Carassius cuvieri* (AP011237.1), *C. gibelio* (JF496198.1), *Chanodichthys ilishaeformis* (NC_029722.1), *Cirrhinus mrigala* (JQ838173.1), *Cyprinus carpio* (X61010.1), *Danio rerio* (AC024175.3), *Hypophthalmichthys molitrix* (EU315941.1), *H. nobilis* (AP011217.1), *Labeo batesii* (AB238967.1), *L. nasus* (AP013333.1), *L. rohita* (AP011201.1), *Megalobrama amblycephala* (AP011219.1) and *M. terminalis* (AB626850.1).
